# Allopurinol reduces antigen-specific and polyclonal activation of human T cells

**DOI:** 10.3389/fimmu.2012.00295

**Published:** 2012-09-21

**Authors:** Damián Pérez-Mazliah, María C. Albareda, María G. Alvarez, Bruno Lococo, Graciela L. Bertocchi, Marcos Petti, Rodolfo J. Viotti, Susana A. Laucella

**Affiliations:** ^1^Instituto Nacional de Parasitología “Dr. Mario Fatala Chaben”Ciudad Autónoma de Buenos Aires, Argentina; ^2^Sección Enfermedad de Chagas, Hospital Interzonal General de Agudos “Eva Perón”, San MartínProvincia de Buenos Aires, Argentina

**Keywords:** allopurinol, T cells, xanthine oxidase, Th1 cytokines, anti-inflammatory

## Abstract

Allopurinol is the most popular commercially available xanthine oxidase inhibitor and it is widely used for treatment of symptomatic hyperuricaemia, or gout. Although, several anti-inflammatory actions of allopurinol have been demonstrated *in vivo* and *in vitro*, there have been few studies on the action of allopurinol on T cells. In the current study, we have assessed the effect of allopurinol on antigen-specific and mitogen-driven activation and cytokine production in human T cells. Allopurinol markedly decreased the frequency of IFN-γ and IL-2-producing T cells, either after polyclonal or antigen-specific stimulation with Herpes Simplex virus 1, Influenza (Flu) virus, tetanus toxoid and *Trypanosoma cruzi*-derived antigens. Allopurinol attenuated CD69 upregulation after CD3 and CD28 engagement and significantly reduced the levels of spontaneous and mitogen-induced intracellular reactive oxygen species in T cells. The diminished T cell activation and cytokine production in the presence of allopurinol support a direct action of allopurinol on human T cells, offering a potential pharmacological tool for the management of cell-mediated inflammatory diseases.

## Introduction

Allopurinol is the most popular commercially available xanthine oxidase inhibitor. This molecule and its *in vivo* active metabolite oxypurinol act as hypoxanthine analogs that irreversibly inhibit xanthine oxidase leading to an inhibition of the catalytic reaction that generates uric acid from hypoxanthine and xanthine, with the concomitant production of superoxide (O_2_^−^) and hydrogen peroxide (H_2_O_2_) (Elion, 1988). Since its approval by the Food and Drug Administration in 1966, allopurinol has been used for treatment of symptomatic hyperuricaemia, or gout (Pacher et al., 2006). However, allopurinol presents a wide range of other potential therapeutic applications such as chronic kidney disease, ischemic reperfusion injury, ischemic heart disease, hypertension, chronic heart failure (Pacher et al., 2006), vascular endothelial dysfunction (George and Struthers, 2009), lens-induced uveitis (Augustin et al., 1994, 1996) and chemotherapy of parasitic infections such as leishmaniasis and Chagas disease (Apt et al., 2005; Harzallah et al., 2010). It was also demonstrated that allopurinol reduces rejection in renal transplant recipients when combined with azathioprine/cyclosporine/prednisolone regimens (Chocair et al., 1993).

Several anti-inflammatory actions of allopurinol have been demonstrated *in vivo* and *in vitro*, probably related with its dose-dependent free radical scavenging ability (Namazi, 2004). Allopurinol decreases the production of Tumor Necrosis Factor-α by human mononuclear cells (Oláh et al., 1994), down-regulates the expression of Intercellular Adhesion Molecule 1 (ICAM-1 or CD54) and P2X_7_ purinergic receptor on human monocytes/macrophages (Mizuno et al., 2004), decreases antigen-specific B cell responses in Ovalbumin-immunized BALB/c mice (Kato et al., 2000) and blocks the induction of Monocyte Chemotactic Factor-1 and Interleukin-6 production in rat vascular smooth muscle cells (Lee et al., 2005). However, the action of allopurinol on T cells is less known. In this regard, allopurinol has been reported to significantly suppress phytohaemagglutin-induced lymphocyte blastogenesis (Kurashige et al., 1985). As allopurinol is a widely prescribed drug with immunomodulatory action and has been proposed as a good candidate for treatment of inflammatory-mediated diseases in which T cells are involved (Grus et al., 2003; Govani and Higgins, 2010), it would be of interest to clarify any putative capacity of allopurinol to modulate T cell activation. Herein, we show that allopurinol diminishes activation as well as cytokine and intracellular reactive oxygen species production by T cells following antigen specific or mitogen-driven stimulation of human peripheral blood mononuclear cells (PBMC).

## Materials and methods

### Selection of study population

Healthy adult volunteers (*n* = 21) aged 27–53 years and asymptomatic chronically *Trypanosoma cruzi*-infected adult subjects (*n* = 7) aged 38–52 years living in urban areas of Buenos Aires, Argentina, were recruited at the Hospital Interzonal General de Agudos “Eva Peron” Buenos Aires, Argentina. Subjects with hypertension, ischemic heart disease, cancer, HIV infection, syphilis, diabetes, arthritis, or serious allergies were excluded from the present study. This protocol was approved by the Institutional Review Board of the Hospital Interzonal General de Agudos “Eva Peron.” Signed informed consents were obtained from all individuals before inclusion in the study, and are stored by the authors.

### Collection of human PBMC

Blood samples were obtained by venipuncture and PBMC were isolated by density gradient centrifugation on Ficoll-hypaque (Amershan, Sweden) and were cryopreserved for later analysis.

### Drugs and antigens

Allopurinol was purchased from USP/BP, Italy. Whole viral particles from Herpes Simplex virus 1 (HSV-1), strain F, were kindly provided by Dr. Carlos A Pujol (Laboratorio de Virología, Departamento de Química Biológica, Universidad de Buenos Aires, Argentina) and obtained as described elsewhere (Matsuhiro et al., 2005). Briefly, HSV-1 strain F was originally obtained from the American Type Culture Collection (Rockville, USA) and propagated in Vero (African green monkey kidney) cells grown in Eagle's minimum essential medium (EMEM, Sigma, USA) supplemented with 1.5% calf serum in 150 cm^2^ cell culture flasks (BD Falcon, USA). Supernatant from HSV-1-infected Vero cell cultures was titrated by plaque formation and used as antigenic stimulation. Supernatant from non-infected Vero cell cultures did not induce T cell responses in ELISPOT assays with PBMC. Peptides derived from Influenza (Flu) virus with high binding affinity for the common class I HLA-supertypes A01, A02, A03, B27, and B35 were synthesized at the University of Georgia Molecular Genetics Instrumentation Facility (Athens, USA). A commercial vaccine (Tetanol Pur, ELEA, Novartis, Germany) was used as an antigen source for tetanus toxoid. An amastigote lysate preparation derived from the Brazil strain of *Trypanosoma cruzi (T. cruzi)* was obtained as previously described (Laucella et al., 2004).

### Determination of allopurinol cytotoxicity

Three ×10^6^ PBMC were incubated in 24-well plates in complete RPMI 1640 10% fetal calf serum in the presence or absence of allopurinol in a concentration range from 25 to 300 μg/ml or drug vehicle (10 mM NaOH) during 2, 24 or 48 h at 37°C. The frequency of total viable and nonviable CD8^+^, CD4^+^, and CD14^+^ cells were determined by staining with 1 μg/ml 7-Amino-actinomycin D (7-AAD) in combination with anti-human CD8 (APC), anti-human CD4 (PE) and anti-human CD14 (FITC) antibodies (Becton Dickinson, USA) for 30 min at 4°C. Cells were then washed, fixed with 2% paraformaldehyde and acquired in a FacsCalibur flow cytometer (Becton Dickinson, USA). Analysis was performed with FlowJo software (Tree Star, USA). At least 5 × 10^5^ events were collected per sample.

### IFN-γ and interleukin 2 enzyme-linked immunosorbent spot (ELISPOT) assays

The number of antigen-specific Interferon-gamma (IFN-γ)-secreting or interleukin-2 (IL-2)-secreting T cells for the different stimuli assessed was determined by *ex vivo* ELISPOT using commercial kits (ELISPOT Human IFN-γ and IL-2 Sets; Becton Dickinson, USA), as described by the manufacturer. Cryopreserved PBMC were seeded in triplicate wells, at a concentration of 4 × 10^5^ cells/well, and were stimulated with HSV-1 (at a multiplicity of infection of 10 plaque forming units/cell), Flu-derived peptide pool (1 μg/ml/peptide), tetanus toxoid (1/20 dilution) or *T. cruzi* lysate (10 μg/ml) in the presence of drug vehicle alone (10 mM NaOH) or allopurinol (300 μg/ml in 10 mM NaOH) for 18–20 h. Stimulation of PBMC with 20 ng/ml Phorbol 12-myristate 13-acetate (PMA, Sigma, USA) plus 500 ng/ml Ionomycin (Sigma, USA) in media was used as positive control of cytokine secretion, while PBMC incubated in media with the addition of allopurinol or drug vehicle served to determine the basal levels of spot-forming cells. For set-up of experimental conditions, 4 × 10^5^ PBMC were stimulated with 100 ng/ml of anti-CD3 in combination with 100 ng/ml of anti-CD28 antibodies (Becton Dickinson, USA) in the presence of drug vehicle alone or different allopurinol concentrations for 18–20 h. Spots were counted and analyzed by Analyzer and ImmunoSpot software (version 6.5; CTL, USA). Responses were considered positive if the number of spot-forming cells in the presence of antigen was at least twice the number of spots in the presence of media, and the number of spots in the latter was less than 10 per 4 × 10^5^ cells. The number of specific IFN-**γ** and IL-2-secreting T cells was calculated by subtracting the value of the wells containing media alone from the antigen/mitogen-stimulated spot count. Only subjects that presented positive antigen-specific responses were included in these assays.

### CD69 surface expression

Cryopreserved PBMC were incubated over night at 37°C in 24-well plates in complete RPMI 1640 10% fetal calf serum at a density of 10^6^ cells/ml. Cells were then stimulated with 1 μg/ml of anti-CD3 in combination with 1 μg/ml of anti-CD28 antibodies (Becton Dickinson, USA) in the presence of drug vehicle alone or 300 μg/ml allopurinol during 5 h. PBMC cultured with complete RPMI in the presence of drug vehicle alone or 300 μg/ml allopurinol served as unstimulated control cells. Then, the cells were washed and stained with anti-human CD4 (PerCP), anti-human CD8 (APC) and anti-human CD69 (PE) (Becton Dickinson, USA) for 30 min at 4°C. At least 5 × 10^5^ events were collected per sample in a FacsCalibur flow cytometer (Becton Dickinson, USA). Analysis was performed with FlowJo software (Tree Star, USA).

### Intracellular reactive oxygen species (iROS) determination

iROS levels were determined as described previously (Yano et al., 1998). Briefly, cryopreserved PBMC were thawed and 1 × 10^6^ PBMC were stained with anti-human CD3 APC (Becton Dickinson, USA) in PBS for 15 min at room temperature. Cells were then washed and further incubated with 100 nM 2′ 7′-dichlorofluorescin di-acetate (DCFH-DA, Sigma, USA) for 15 min at 37°C and shaking, with or without 300 μg/ml allopurinol in PBS. In this case, allopurinol was directly dissolved into PBS to avoid the use of drug vehicle. Thereafter, 100 nM PMA or PBS alone was added to the culture for additional 1hr at 37°C under shaking. Cells were acquired in a FacsCalibur flow cytometer (Becton Dickinson, USA) and analyzed with FlowJo software (Tree Star, USA). At least 2 × 10^5^ events were collected per sample. The intracellular hydrolyzed and oxidized form, 2′ 7′-dichlorofluorescein (DCF), was detected at the FL-1 channel. DCF mean fluorescence intensity represents a measure of iROS.

### Statistical analysis

The Kruskal-Wallis nonparametric test was used to compare the percentages of surface markers expression and 7-AAD incorporation in PBMC treated with different concentrations of allopurinol or drug vehicle. The Wilcoxon signed rank test was applied to compare the frequencies and sizes of spot-forming cells, CD69 expression and iROS production between untreated and allopurinol-treated PBMC. Differences were considered to be statistically significant at *P* < 0.05.

## Results

### *In vitro* treatment with allopurinol exerts no cytotoxicity and has no influence on CD4, CD8, and CD14 expression

**Figure 1:**
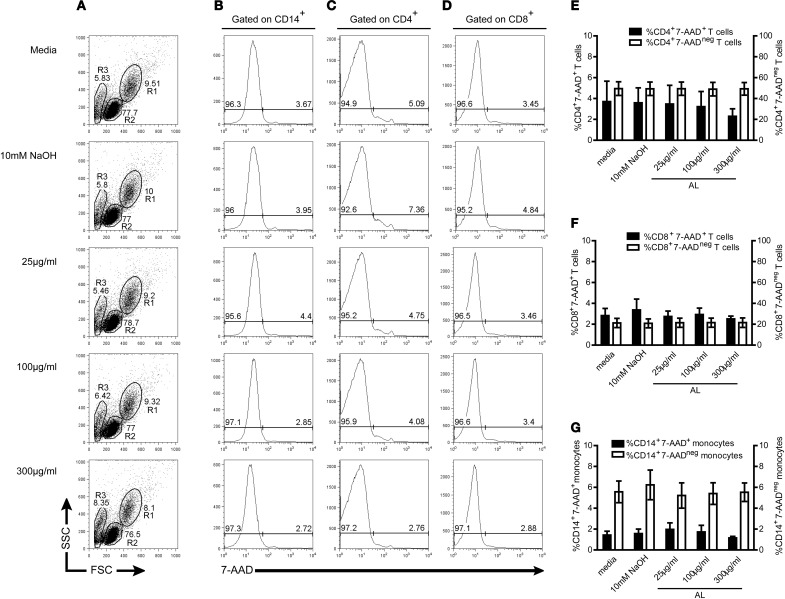
***In vitro* treatment with allopurinol exerts no cytotoxicity and has no influence on CD4, CD8, and CD14 constitutive expression.** PBMC from healthy donors were isolated by density gradient centrifugation on Ficoll-hypaque. PBMC were incubated in the presence or absence of allopurinol (25–300 μg/ml) or drug vehicle (10 mM NaOH) during 48 h and analyzed by four color flow cytometry. The frequency of total non-viable and viable CD14^+^, CD4^+^, and CD8^+^ cells were determined by staining with 1 μg/ml 7-aminoactinomycin D (7-AAD) in combination with CD14 (FITC), CD4 (PE), and CD8 (APC) antibodies. **(A–D)** Representative gate selection and analysis for monocytes and T cells. Monocytes (R1) and lymphocytes (R2) were gated by forward and side light scatter **(A)**, including an area generally enriched in death cells (R3). CD14^+^, CD4^+^, or CD8^+^ cells were selected, and from these populations 7-AAD histograms were made to determine the percentages of 7-AAD^+^ (on the right) and 7-AAD^−^ (on the left) cells on total CD14^+^ **(B)**, CD4^+^ **(C)** or CD8^+^ **(D)** T cells. Mean percentages ± SEM of CD4^+^7-AAD^+^ **(E)**, CD8^+^7-AAD^+^ **(F)** or CD14^+^7-AAD^+^ **(G)**, (left Y axis, black bars) and CD4^+^7-AAD^−^ **(E)**, CD8^+^7-AAD^−^ **(F)** or CD14^+^7-AAD^−^ **(G)**, (right Y axis, white bars) from 5 samples assessed. *P* > 0.05 among different allopurinol concentrations, drug vehicle, and media alone, as determined by Kruskal–Wallis test. AL, allopurinol.

In order to rule-out any possible cytotoxic effect of allopurinol on human PBMC, cell viability was evaluated by staining with 7-AAD, a nucleic acid fluorescent dye that penetrates the cell membrane of nonviable cells and allows the quantification of dying or dead cells by flow cytometry. Allopurinol did not affect either cell viability (Figures 1A–D) or the constitutive expression of CD4, CD8, and CD14, regardless the dose (Figures 1E–G) in a 48 h assay. Neither significant difference in cell viability or in the expression of surface markers was recorded after 2 or 24 h-incubation in the presence of allopurinol (data not shown).

### Allopurinol decreases polyclonal production of IFN-γ and IL-2 by human PBMC

**Figure 2:**
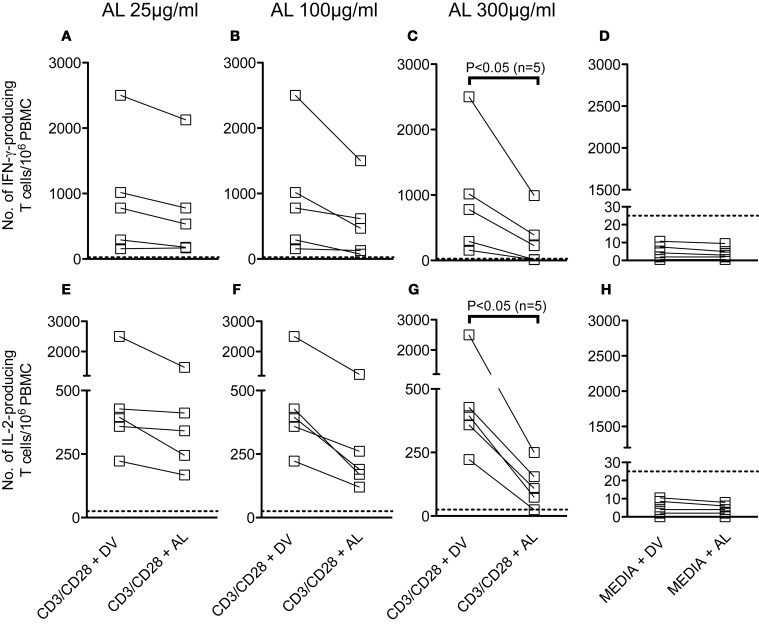
**Effect of different concentrations of allopurinol on polyclonal activation-driven IFN-γ and IL-2 production by human T cells.** PBMC from healthy donors were stimulated with anti-CD3 (100 ng/ml) plus anti-CD28 (100 ng/ml) antibodies, or remained with media alone, in the presence of different concentrations of allopurinol (+ AL) or drug vehicle (+ DV), for 18–20 h and the number of IFN-γ-secreting **(A–D)** or IL-2-secreting **(E–G)** T cells were determined by *ex vivo* ELISPOT assays. **(D** and **H)** The samples were cultured in media with the addition of AL or DV. Each line represents an individual subject. Panels **A–C** and **E–G** show the net number of spot-forming cells (spot-forming cells in media were subtracted). Dotted lines represent the threshold for positive IFN-γ or IL-2 ELISPOT responses, as defined in Materials and Methods. Comparisons between T cell responses in the absence or presence of allopurinol upon stimulation were performed by Wilcoxon signed rank test.

We have evaluated the effect of allopurinol on the ability of human T cells to secrete IFN-γ and IL-2 after stimulation with different pathogen-specific antigens. Considering that previous studies have shown that treatment of human PBMC with allopurinol in a range of 25–100 μg/ml impairs several monocyte functions (Mizuno et al., 2004), experimental conditions for cytokine secretion by the ELISPOT technique were set-up by stimulation of PBMC with anti CD3/CD28 antibodies in the presence of 25–300 μg/ml allopurinol. Although decreases in both IFN-γ (Figures 2A–C) and IL-2 (Figures 2E–G) production were already observed at 100 μg/ml allopurinol, these decreases became significant with 300 μg/ml allopurinol. Thus, the latter concentration was chosen for further studies. Drug vehicle alone did not exert any effect on cytokine production (Figures 2D and H).

### *In vitro* treatment with allopurinol attenuates activation-driven CD69 expression in human T cells

**Figure 3:**
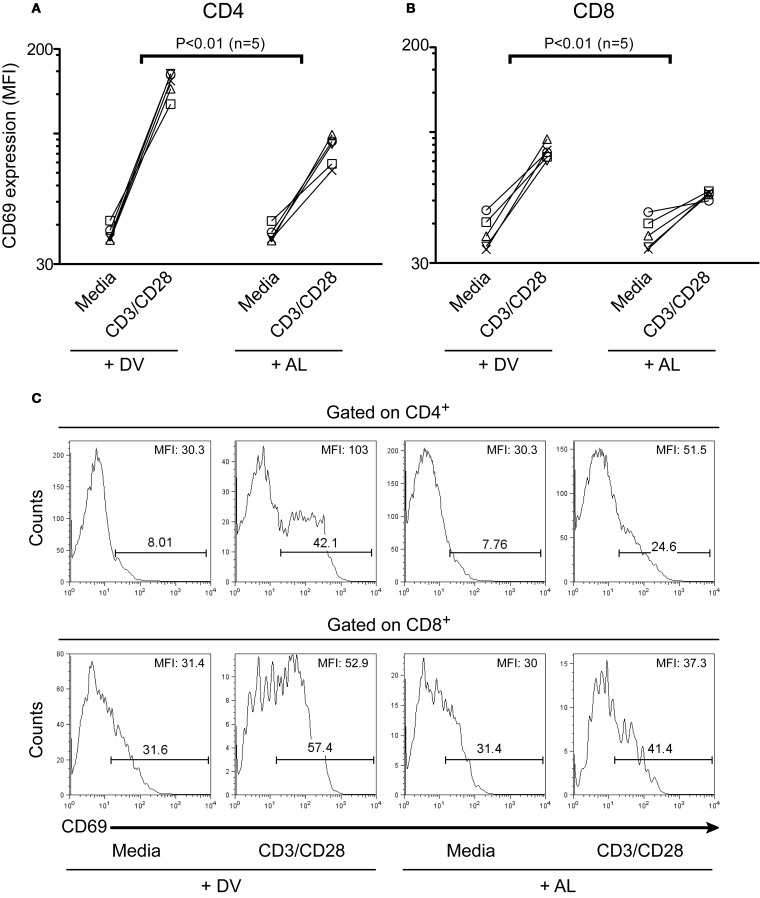
**Allopurinol attenuates activation-driven CD69 upregulation in CD4^+^ and CD8^+^ T cells.** PBMC were incubated for 5 h with media or with anti-CD3 (1 μg/ml) plus anti-CD28 (1 μg/ml) antibodies, in the presence of allopurinol (+ AL, 300 μg/ml) or drug vehicle alone (+ DV). Cells were then stained with anti-CD4 (PerCP), anti-CD8 (APC) and anti-CD69 (PE) and analyzed with four color flow cytometry. Lymphocytes were gated by forward and side light scatter. From this population single color CD4 or CD8 staining histogram was made, and the expression of CD69 was analyzed. Each line shows the mean fluorescence intensity (MFI) of CD69 expression on CD4^+^ **(A)** or CD8^+^ **(B)** T cells before and following stimulation with anti-CD3/CD28 antibodies, in the presence of drug vehicle (+ DV) or allopurinol (+ AL), for each subject evaluated. Comparisons between the differences in net CD69 expression (subtracting CD69 MFI in media) with and without allopurinol were performed by Wilcoxon signed rank test. Representative histogram plots of CD69 expression on CD4^+^ (**C**, top panels) and CD8^+^ (**C**, bottom panels) T cells with the indicated treatment. The numbers over the bars indicate the percentages of CD69^+^ cells and the numbers in the top right corner indicate the MFI of CD69 expression on CD4^+^ (top) and CD8^+^ (bottom) T cells.

CD69 is a cell surface molecule upregulated early after T cell activation (Hara et al., 1986; Cosulich et al., 1987; Risso et al., 1989). In this study, we also evaluated the effect of allopurinol on early events of human T cell activation by measuring alterations in CD69 expression. As shown in Figure 3, allopurinol attenuates CD69 upregulation on CD4^+^ (Figure 3A and top panels Figure 3C) and CD8^+^ (Figure 3B and bottom panels Figure 3C) T cells after anti-CD3/CD28 stimulation. The mean fold increase in CD69 expression after anti-CD3/CD28 stimulation was 38% and 30% lower in the presence of allopurinol for CD4^+^ and CD8^+^ T cells, respectively, compared with drug vehicle alone. Conversely, neither allopurinol nor the drug vehicle altered the expression of CD69 on unstimulated T cells after the 5 h-incubation period (Figures 3A–C).

### *In vitro* treatment with allopurinol reduces iROS production in human T cells

**Figure 4:**
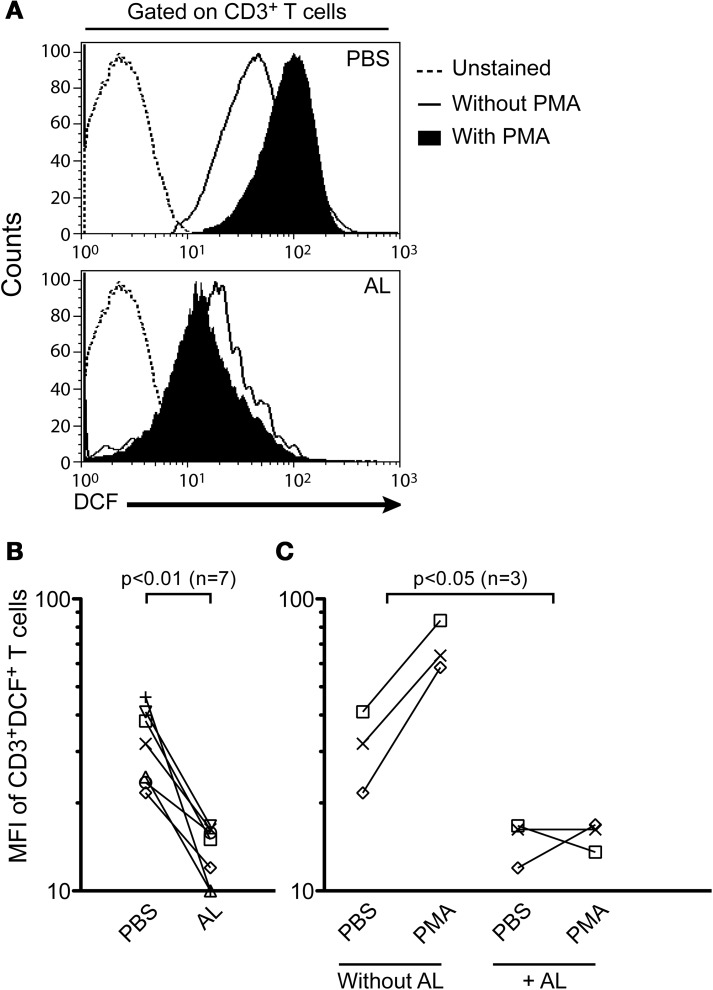
**Allopurinol attenuates spontaneous and mitogen-activated iROS production in human T cells.** PBMC were stained with anti-CD3 PE and incubated at 37°C for 1 h with DCFH-DA and PBS alone or with PMA (100 nM) in the presence (+ AL, 300 μg/ml) or absence of allopurinol. The oxidized form, DCF represents a measure of iROS. Lymphocytes were gated by forward and side light scatter. From this population single color CD3 staining histogram was made, and the expression of DCF in the gated population was analyzed. **(A)** Representative iROS expression on CD3^+^ T cells in the absence (upper panel) or presence (bottom panel) of allopurinol. **(B)** Cumulative data of spontaneous iROS production from 7 subjects. Each line represents spontaneous iROS levels for each patient in the absence (PBS) or in the presence (AL) of allopurinol. **(C)** Cumulative data of induced iROS production. Each line show iROS release for each subject prior and following stimulation with PMA in the presence (+ AL) or absence of allopurinol. Comparisons were performed by Wilcoxon signed rank test. MFI: mean fluorescence intensity.

T cells increase the production of ROS early after activation and their effector function is strongly regulated by ROS, through distinct T cell receptor (TCR) pathways (Devadas et al., 2002; Williams and Kwon, 2004; Yan and Banerjee, 2010). Taking into account that allopurinol is a potent scavenger of ROS (Moorhouse et al., 1987) and presents antioxidant activities *in vivo* (Augustin et al., 1994), we hypothesized that allopurinol action on T cell activation could be explained by its scavenging action during early activation events of T cells. We therefore studied the allopurinol action on iROS production in human T cells by measuring DCF mean fluorescence intensity in response to PMA, a known stimulus for iROS production in human T cells (Yano et al., 1998). Allopurinol significantly reduced either the levels of spontaneous (Figures 4A and B) or PMA-induced (Figures 4A and C) iROS in total T cells.

### Allopurinol decreases antigen-specific IFN-γ and IL-2-producing T cell responses in human PBMC

**Figure 5:**
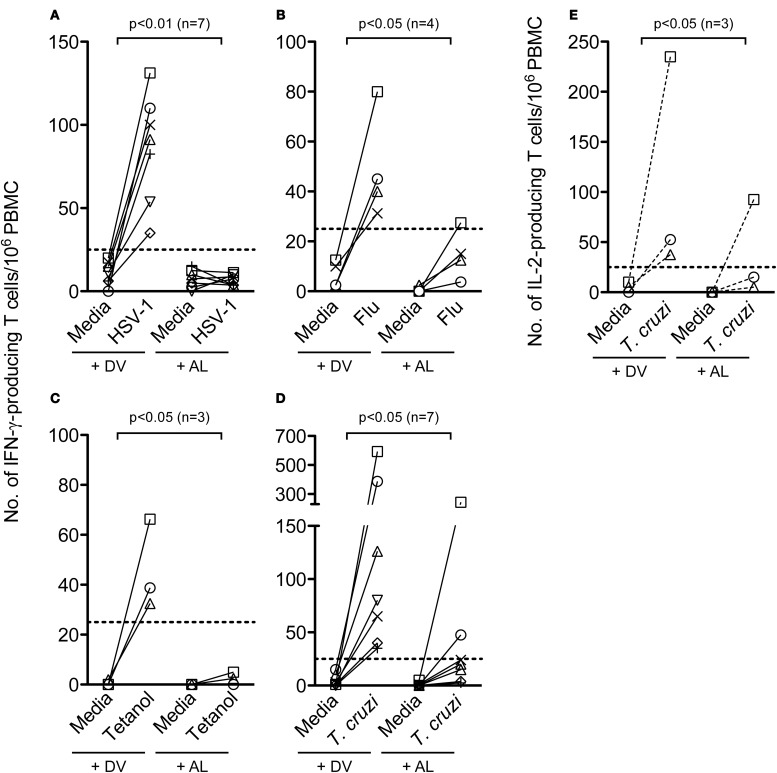
**Allopurinol attenuates antigen-specific activation-driven IFN-γ and IL-2 production by human T cells.** PBMC from healthy donors were stimulated with full viral particles from HSV-1 **(A)**, a Flu-derived peptide pool **(B)**, tetanus toxoid **(C)**, or cultured in the absence of antigen stimulation (media), in the presence of allopurinol (+ AL, 300 μg/ml) or drug vehicle alone (+ DV) for 18–20 h, and the number of IFN-γ-secreting or IL-2-secreting **(E)** T cells were determined by *ex vivo* ELISPOT assays. PBMC from a group of chronically *T. cruzi*-infected subjects were stimulated with a *T. cruzi*-derived amastigote lysate **(D** and **E)**. Each line represents an individual subject. Dotted lines represent the threshold for positive T cell responses, as defined in Materials and Methods. Comparisons between T cell responses in the absence or presence of allopurinol upon stimulation were performed by Wilcoxon signed rank test.

**Figure 6:**
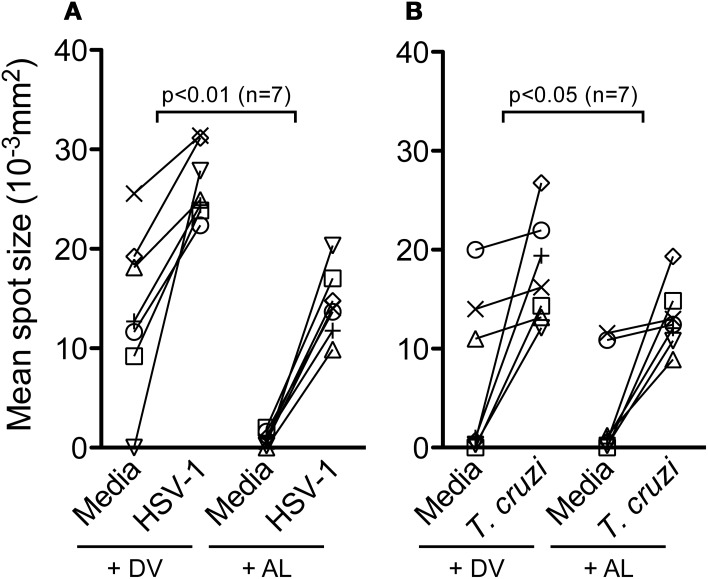
**Allopurinol decreases IFN-γ secretion at a single cell level.** IFN-γ ELISPOT assays were performed by stimulation, or not (media), of PBMC from healthy donors with full viral particles from HSV-1 **(A)**, or PBMC from a group of chronically *T. cruzi*-infected subjects with a *T. cruzi*-derived amastigote lysate **(B)**, in the presence of allopurinol (+ AL, 300 μg/ml) or drug vehicle (+ DV), for 18–20 h. Mean spot sizes represent an estimate of IFN-γ production per cell. Each line represents an individual subject. Comparisons between T cell responses in the absence or presence of allopurinol upon stimulation were performed by Wilcoxon signed rank test.

The effect of allopurinol on antigen-specific cytokine production by T cells was also evaluated. To achieve this aim, T cell responses specific for pathogen-derived antigens representative of different infection models, including a chronic viral infection (HSV-1), a solved viral infection (Flu) and a parasitic chronic infection (*T. cruzi*) were measured ex vivo by IFN-γ and IL-2 ELISPOT assays. T cell responses specific for Tetanus toxoid were also analyzed in a group of vaccinated subjects. Allopurinol markedly decreased the frequency of IFN-γ-producing T cells independently of the antigen used as stimuli, including full viral particles from HSV-1, a Flu-derived peptide pool with high binding affinity for common class I HLA-supertypes, tetanus toxoid and a *T. cruzi* lysate preparation (Figures 5A–D). Likewise, IL-2 production was also diminished in the presence of allopurinol upon stimulation with *T. cruzi* antigens (Figure 5E). The mean spot size of IFN-γ was significantly decreased in the presence of allopurinol, indicating not only a reduction in the frequency of IFN-γ-producing T cells, but also in cytokine production at a single cell level (Figures 6A and B). As seen after polyclonal stimulation, drug vehicle alone did not have any effect on cytokine production after antigen-specific stimulation (Figures 5A–E and Figures 6A and B).

## Discussion

Xanthine Oxidase inhibitors have emerged as therapeutic tools for inflammatory-mediated diseases, including those in which T cells are involved (Grus et al., 2003; Pacher et al., 2006; Govani and Higgins, 2010; Ng et al., 2011; Sliem and Nasr, 2011). In the present study, the effect of allopurinol on late (i.e., cytokine production) as well as on early (i.e., CD69 expression and iROS production) events of human T cell activation was investigated. We show, for the first time, that allopurinol decreases class I and class II-restricted antigen-specific and polyclonal IFN-γ and IL-2 production, in addition to the expression of activation markers by human T cells.

Antigen-specific activation of T cells requires recognition of peptide-MHC complexes by the TCR on antigen presenting cells, costimulation and the expression of adhesion molecules. The recognition of antigen by the TCR initiates transcriptional activation of particular genes that mediate T cell responses. We found that allopurinol decreased IFN-γ production by T cells specific for different pathogens probably due to a direct effect of the drug on T cells as well as on antigen-presenting cells. This view is supported by other findings showing that allopurinol downregulates the expression of ICAM-1 on monocytes (Mizuno et al., 2004) and modulates protein kinase C activity (Kang et al., 2006), one of the main enzymes involved in biochemical pathways inducing T cell responses. IFN-γ secretion was also reduced in the presence of allopurinol after polyclonal stimulation of T cells with anti-CD3/CD28 that mimics signals generated by the TCR complex, bypassing the need for T cell-antigen presenting cell interaction, supporting that allopurinol might exert a direct action on T cells. Moreover, we showed that IL-2 production, a cytokine that drives T cell activation, is reduced in the presence of allopurinol. The lower amounts of antigen-specific cytokine production, as evidenced by the smaller spot size and the attenuation of CD69 upregulation upon activation with CD3/CD28, further support the specific effect of allopurinol on T cell activation.

Diverse evidences have shown that the redox state can influence T cell function *in vitro* and *in vivo* (Griffiths et al., 2011). Cross-linking of the TCR and the co-stimulatory molecule CD28 in human T cells results in enhanced iROS production that is needed for NF-κB and IL-2 expression (Los et al., 1995) and is consistent with an important role for ROS in the immediate early events during activation (Yan and Banerjee, 2010). Lipid metabolism, mitochondria, and/or NADPH oxidases have been claimed to be the source of iROS during T cell activation (Williams and Kwon, 2004). Although xanthine oxidase also mediates ROS generation, this enzyme is not expressed by human immune cells (Dröge, 2002). Xanthine oxidase is distributed in the liver, gut, lung, kidney, heart, and brain, accounting for only a minor proportion of total ROS production under normal conditions (Dröge, 2002). Thus, the modulation of T cell function observed herein would be accounted for the ROS scavenging action of allopurinol but not for an inhibition in ROS production mediated by the xanthine oxidase enzyme. Thus, allopurinol would deprive T cells from iROS which are required during the early activation of the NF-kB complex (Los et al., 1995) and PKC (Kang et al., 2006), thus promoting a hyporesponsive state of T cells. In agreement with this notion, allopurinol has been recently shown to reduce NF-κB pathway activation, pro-inflammatory cytokines production, and oxidative stress in different *in vivo* models of inflammation (Correa-Costa et al., 2011; Aldaba-Muruato et al., 2012; Demirel et al., 2012).

Both T cells and dendritic cells elevate the intracellular oxidation status upon antigen-specific interaction, and bidirectional dendritic cell-T cell communication can be blocked by interfering with redox regulation pathways (Matsue et al., 2003). Moreover, alteration in redox status of T cells and dendritic cells has been recently proposed as a putative mechanism of regulatory T cells action (Yan et al., 2010). Although, we cannot rule out the possibility that the effect of allopurinol on antigen-specific T cell responses is due to, at least partially, an impairment in antigen presentation by the action of allopurinol on antigen-presenting cells, the reduction of iROS production by PMA-stimulated T cells in the presence of allopurinol for short periods of time further supports the idea of a direct action of allopurinol on T cell activation. Future studies regarding the effect of allopurinol on the expression of costimulatory molecules, as well as studies at the transcriptional level on T cells, will help us to further clarify these ideas.

CD4^+^ IFN-γ-producing T_h1_ cells have long been associated with the pathogenesis of many organ-specific autoimmune diseases (Dardalhon et al., 2008). Herein, we report that these responses might be inhibited by allopurinol, *in vitro*. A beneficial effect of allopurinol on cell-mediated diseases is also supported by an improvement of the response to thiopurine treatment by the addition of allopurinol in individuals with inflammatory bowel disease, a T_h1_-polarized disease (Gardiner et al., 2011; Smith et al., 2012). Inflammation has also been pointed out as a potential target for therapy of chronic heart failure (Celis et al., 2008), that has been strongly correlated with T cell activation and altered T_h1_/T_h2_ balance (Cheng et al., 2009). iROS production by phagocytic leukocytes is also increased in heart failure patients (Castro et al., 2003) and it is a major cause of endothelial dysfunction as iROS can act both, as triggers or amplifiers of the inflammatory response (Deschamps and Spinale, 2006; Castro et al., 2008). Several studies have demonstrated that treatment with xanthine oxidase inhibitors of patients suffering from heart failure resulted in reduced oxidative stress and improved endothelial function (Landmesser et al., 2002; Castro et al., 2005; Hare et al., 2008). Our results showed that allopurinol decreases iROS production by T cells which might have important therapeutic implications since down-regulation of inflammatory T cells is the treatment of choice for many inflammatory diseases. In agreement with this notion, the use of a different immunomodulating agent, Pentoxifylline, improved the clinical status of patients with idiopathic-dilated and ischemic cardiomyopathy (Barnett and Touchon, 1990; Skudicky et al., 2000; Sliwa et al., 2002, 2004).

Allopurinol is rapidly absorbed *in vivo*, reaching peak plasma concentrations within 30–60 min, following oral administration (Pea, 2005). Allopurinol has relatively short half-life in plasma (2–3 h) because it is rapidly metabolized *in vivo* and converted almost completely into to the oxidized metabolite, oxypurinol, which has the same therapeutic pattern but a much longer elimination half-life (14–30 h) than the parent compound (Pea, 2005). Allopurinol is negligibly bound to plasma proteins, and gets spread to different tissues, including vascular tissue, liver, intestine, and heart (Murrell and Rapeport, 1986). Therefore, the maximum possible concentration of allopurinol *in vivo* is hard to estimate, making it difficult to predict the *in vivo* physiological relevance of drug concentrations applied, *in vitro*, in the present study. Moreover, it has been demonstrated that allopurinol can be differentially metabolized *in vitro* and *in vivo* (Kramer and Feldman, 1977).

Determination of plasma concentrations of oxypurinol highly differs depending on the population studied, the dose and the route of administration, the length of treatment and the time-points after drug intake chosen for measurements (Rodnan et al., 1975; Breithaupt and Tittel, 1982; Berlinger et al., 1985; Murrell and Rapeport, 1986; Emmerson et al., 1987; Day et al., 1988a,b; Graham et al., 1996; Turnheim et al., 1999; Guerra et al., 2001; Kaya et al., 2006; Panomvana et al., 2008; Stocker et al., 2008; Van Dijk et al., 2008; Torrance et al., 2009; Stamp et al., 2011). For instance, Van Dijk et al. demonstrated that allopurinol reaches a mean maximal plasma concentration of 41.90 μg/ml within minutes after a dose of 15 mg/kg body weight administered i.v. in pregnant sows (Van Dijk et al., 2008). In a survey studying 50 adult patients receiving variable daily doses of allopurinol, with 83% of the patients taking 300 mg/day, during 6 ± 7.5 years on average, a wide range of plasma oxypurinol concentration (i.e., from 2.8 to 55.8 μg/ml) was observed (Day et al., 1988b). Differences in the levels of serum oxypurinol from patient to patient taking the same dose of allopurinol may result from differences in absorption, rate of metabolism, xanthine oxidase levels, the amount of drug bound to xanthine oxidase in the tissues and/or renal function. Even diet and exercise can significantly alter the pharmacokinetics of oxypurinol (Berlinger et al., 1985; Kaya et al., 2006).

In the present work, we observed that a single dose of 100 μg/ml allopurinol decreased T cell production of cytokines within hours after *in vitro* culture, but reaching a statistical significant reduction upon treatment with 300 μg/ml of allopurinol. As oxypurinol accumulates and reaches a steady-state level during long-term administration (Murrell and Rapeport, 1986), the effect of allopurinol observed within hours after *in vitro* culture is probably only comparable to the effect observed in subjects receiving long-term treatment with allopurinol. In this regard, allopurinol is generally well tolerated, allowing long-term treatments and administration of high doses (Momeni and Aminjavaheri, 1995; Momeni et al., 2002).

Allopurinol, administered at 600 mg/day during 3 months, constitutes a second line drug for the treatment of chronic Chagas disease, caused by *Trypanosoma cruzi* infection. Allopurinol was also shown to be safe and effective in the treatment of Chagas disease reactivation after heart transplantation (Almeida et al., 1996; Bestetti and Theodoropoulos, 2009). In a recent pilot study of a sequential combined treatment with allopurinol and benznidazole in the chronic phase of *Trypanosoma cruzi* infection (Pérez-Mazliah et al., 2012), we have observed that the frequency of peripheral naïve CD4^+^ and CD8^+^ T cells, that are generally diminished in this phase of the infection (Albareda et al., 2006, 2009), is improved along with a decrease in the frequency of peripheral CD4^+^ and CD8^+^ T cells expressing the activation marker HLA-DR after completion of allopurinol administration. These changes were sustained following the consecutive treatment with benznidazole, supporting the immunomodulating activity of allopurinol, *in vivo*, on human T cells.

Allopurinol hypersensitivity syndromes, like the Stevens-Johnson syndrome, is an infrequent but life-threatening adverse effect that affects about 0.4% patients receiving allopurinol therapy (Arellano and Sacristán, 1993; Pluim et al., 1998) and whose mechanisms remain unclear. Three potential factors involved in allopurinol hypersensitivity syndromes are the genetic background, dose accumulation, and immunological responses to the drug. However, a relationship between the appearance of allopurinol hypersensitivity syndromes and the use of high doses has not been demonstrated (George et al., 2006). Risk factors for development of allopurinol hypersensitivity syndromes include aging, renal impairment, diuretic use, and some ethnics groups (Chinese descent) (Lee et al., 2008). In the same pilot study mentioned above (Pérez-Mazliah et al., 2012), the use of allopurinol in doses of 600 mg/day for 90 consecutive days was very well tolerated and we did not observe any case of severe adverse reaction. This observation is in agreement with previous studies (Gallerano et al., 1990; Momeni and Aminjavaheri, 1995; Apt et al., 1998; Momeni et al., 2002; Apt et al., 2003, 2005), in which doses even higher than 1 g/day of allopurinol during months were administered without registering high incidence of adverse effects.

The immunomodulatory action of allopurinol described in the present work in combination with previous observations could raise the idea that allopurinol would favor the occurrence of opportunistic infections. We have not observed a higher incidence of opportunistic infections in chronically *Trypanosoma cruzi*-infected patients under treatment with doses of 600 mg per day of allopurinol during 3 months (Pérez-Mazliah et al., 2012). This is again in accordance with previous observations, even in long-term and high dose treatments with allopurinol (Gallerano et al., 1990; Momeni and Aminjavaheri, 1995; Apt et al., 1998, 2003, 2005; Momeni et al., 2002). Thus, the immunomodulatory effect of allopurinol does not seem to generate the level of immunosuppression required to favor opportunistic infections *in vivo*, at least in the context of the studies cited herein.

Nonetheless, due to its low frequent but potentially severe side effects as well as its immunomodulatory action, it is highly recommended a close follow-up of patients receiving allopurinol throughout the entire duration of the treatment. Particular attention should be paid to those populations at high risk of developing side effects and those presenting alterations in normal immune system function. In summary, this study raise evidence in line with an immunomodulatory action of allopurinol on human T cells, offering a potential pharmacological tool for the management of cell-mediated inflammatory diseases.

### Conflict of interest statement

The authors declare that the research was conducted in the absence of any commercial or financial relationships that could be construed as a potential conflict of interest.

## Abbreviations

7-AAD: 7-aminoactinomycin D
AL: allopurinol
DCF: 2′ 7′-dichlorofluorescein
DCFH-DA: 2′ 7′-dichlorofluorescin di-acetate
DV: drug vehicle
HSV-1: herpes simplex virus-1
iROS: intracellular reactive oxygen species
PBMC: peripheral blood mononuclear cells
PMA: Phorbol 12-myristate 13-acetate
*T. cruzi*: *Trypanosoma cruzi*.
